# On the probability of lymph node negativity in pN0-staged prostate cancer—a theoretically derived rule of thumb for adjuvant needs

**DOI:** 10.1007/s00066-021-01841-x

**Published:** 2021-09-02

**Authors:** Frank Paulsen, Jens Bedke, Daniel Wegener, Jolanta Marzec, Peter Martus, Dominik Nann, Arnulf Stenzl, Daniel Zips, Arndt-Christian Müller

**Affiliations:** 1 Department of Radiation Oncology, Eberhard Karls University, Hoppe-Seyler-Str. 3, 72076 Tübingen, Germany; 2 Department of Urology, Eberhard Karls University, Hoppe-Seyler-Straße 3, 72076 Tübingen, Germany; 3 Institute for Clinical Epidemiology and Applied Biometry, Eberhard Karls University, Silcherstraße 5, 72076 Tübingen, Germany; 4 Institute of Pathology, Eberhard Karls University, Liebermeisterstr. 8, 72076 Tübingen, Germany

**Keywords:** Sensitivity, Nodal metastasis, Prediction model, Negative predictive value, Whole-pelvis radiotherapy

## Abstract

**Purpose:**

The extent of lymphadenectomy and clinical features influence the risk of occult nodes in node-negative prostate cancer. We derived a simple estimation model for the negative predictive value (npv) of histopathologically node-negative prostate cancer patients (pN0) to guide adjuvant treatment.

**Methods:**

Approximations of sensitivities in detecting lymph node metastasis from current publications depending on the number of removed lymph nodes were used for a theoretical deduction of a simplified formulation of npv assuming a false node positivity of 0.

**Results:**

A theoretical formula of npv = p(N0IpN0) = (100 − prevalence) / (100 − sensitivity × prevalence) was calculated (sensitivity and preoperative prevalence in %). Depending on the number of removed lymph nodes (nLN), the sensitivity of pN0-staged prostate cancer was derived for three sensitivity levels accordingly: sensitivity = f(nLN) = 9 × nLN /100 for 0 ≤ nLN ≤ 8 and f(nLN) = (nLN + 70) /100 for 9 ≤ nLN ≤ 29 and f(nLN) = 1 for nLN ≥ 30.

**Conclusion:**

We developed a theoretical formula for estimation of the npv in pN0-staged prostate cancer patients. It is a sine qua non to use the formula in a clinically experienced context before deciding to electively irradiate pelvic lymph nodes or to intensify adjuvant systemic treatment.

## Introduction

The extent of lymphadenectomy during the course of radical prostatectomy is adapted to clinical risk factors. In general, limited, i.e., standard lymph node dissection (sLND) is associated with an underestimation of the true number of pathologic nodes, especially in patients with upstaging of pretherapeutic risk factors [1]. In contrast to sLND, extended lymph node dissection (eLND) is able to detect additional, i.e., occult metastases in approximately 5–6% of low risk, 20–25% of intermediate risk, and 30–40% of high-risk prostate cancer patients [1]. Furthermore, prostate cancer-specific survival (PCSS) improves with the extent of LND [2].

However, detection of occult lymph node metastases is associated with a decreased survival probability, leading to an outcome comparable to node-positive disease [3]. Otherwise, patients with lymph node metastases (pN1) receiving adjuvant treatment have significantly improved cancer-specific and overall survival. This benefit was especially evident in patients receiving radiotherapy in addition to androgen deprivation therapy (ADT) with an overall survival of 74% at 10 years for combined therapy, compared to 55% with ADT alone [4]. However, even in pN0-staged patients, an estimation of the negative predictive value (npv) seems to be necessary for the decision regarding adjuvant strategies including dose-escalated RT of the prostatic fossa, whole-pelvis radiotherapy (WPRT) of the lymphatics, or ADT [5–7].

Several models or nomograms using pretherapeutic characteristics such as T‑stage, prostate-specific antigen (PSA), Gleason score (GS), and number of positive cores were established to calculate the prevalence of N+-stage [8–12]. The risk of being N1 is dependent on the prevalence of lymph node metastasis. In general, histopathological involved nodes (pN1) are staged correctly. The probability of a false-positive diagnosis of pN1 in the case of true N0 seems to be negligible. The risk of true N1 in case of pN0 is furthermore dependent on the number of removed and pathologically examined lymph nodes, potentially due to a geographic miss during resection or atypical localization of individual lymph nodes [1, 13, 14]. There are published estimations on the risk of true N+-stage despite pN0 stage with complex and confusing npv estimations [14]. To identify pN0 patients with a risk for residual nodes, we mathematically derived a simple formula for the npv. This tool provides the clinician with a simple preliminary estimation of a patient’s chance of true N0 stage (npv) after surgically and pathologically confirmed pN0 stage to guide adjuvant treatment in pN0-staged prostate cancer patients in cases of increased individual risk.

## Patients and methods

### Patient data

Current literature was searched for large series on radical prostatectomy (*n* > 3000) and the terms “prostate cancer” and “lymph node metastasis” with information about sensitivity of lymphadenectomy dependent on the number of removed lymph nodes. Three publications were eligible for model development fulfilling the mentioned preconditions (Table 1).

**Table 1:** Publications used for estimation of the sensitivity of lymphadenectomy dependent on the number of removed lymph nodes

Author	Patients	Removed LN	Pts	pN+ in	pN+ in	pN+ in
	*n*, Origin of data	(*n* : median (range))	pN+ (*n*)	LR (*n*)	IR (*n*)	HR (*n*)
Abdollah [13]	20789, SEER	5 (1–40)	529	8	169	352
Kluth dev [14]	7135, 8 academic centers	6 (1–77)	415	Ns	Ns	Ns
Kluth val [14]	4209, single center	16 (5–66)	564	Ns	Ns	Ns
Rieken val [15]	50598, SEER	5 (1–89)	1578	Ns	Ns	Ns

*LN* lymph nodes, *n* number, *pN+* pathologically diagnosed LN metastasis, *LR* low risk, *IR* intermediate risk, *HR* high risk, *dev* development cohort, *val* validation cohort, *SEER* Surveillance, Epidemiology and End Results database, *Ns* not specified

Abdollah et al. demonstrated the “probability of correctly staging patients with lymph node metastases”, interpreted as f(nLN) = sensitivity of the whole procedure (staging, surgery, and pathology: pN0), dependent on the number of removed lymph nodes in 20,789 patients [13]. Kluth et al. described the “probability of missing nodal disease” defined as function g(nLN) = 1 — sensitivity in a development cohort (7135 patients) and a validation cohort (4209 patients), again dependent on the number of removed lymph nodes [14]. Rieken et al. (2017) performed an external validation of this dataset in 50,598 patients [15].

### Statistics

Curve fit was done using splines with two domains of definition for nLN below a sensitivity of 100%. A simple formulation was deduced after calculation of the conditional probability for true negativity in case of pN0-staged (npv) prostate cancer. False positivity (fp) is clinically negligible and therefore set to be 0 (fp = 0). Fits and examples were performed with Excel 2010 (Microsoft Corporation, Washington, USA). Color artwork was conducted using Canvas X (ACD Systems International Inc., British Columbia, Canada).

## Results

### Development of a simple model for npv

Based on the formula by Bayes [16], the following deduction was made assuming a false-positive (fp) test value of 0 for standard histology, i.e., specificity = 1:

Bayes’ formula:

$$npv=\frac{\text{specificity }\mathrm{x}\left(1-\text{prevalence}\right)}{\text{prevalence }\mathrm{x}\left(1-\text{sensitivity}\right)+\text{specificity }\mathrm{x}\left(1-\text{prevalence}\right)}$$

Specificity is set to be 1:

$$npv=\frac{1\mathrm{x}\left(1-\text{prevalence}\right)}{\text{prevalence }\mathrm{x}\left(1-\text{sensitivity}\right)+1\mathrm{x}\left(1-\text{prevalence}\right)}$$

$$npv=\frac{1-\text{prevalence}\,}{1-\left(\text{sensitivity }\mathrm{x}\text{ prevalence}\right)}$$

**(I) npv in %:**

$$npv=\frac{100-\text{prevalence}\,}{100-\left(\text{sensitivity }\mathrm{x}\text{ prevalence}\right)}$$

The sensitivity as a function of the number of removed lymph nodes, f(nLN), is derived in the following section (see formula II). This npv formulation is of general validity with the precondition of negligible fp (fp = 0).

### Fit curve for npv formulation

**Fig. 1:**
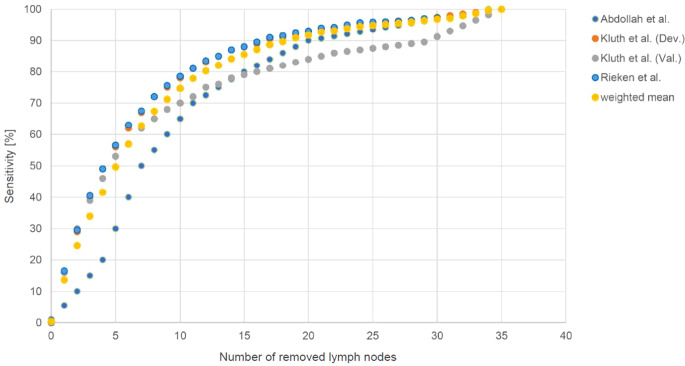
Sensitivity of lymphadenectomy taken from published data (weighted mean sensitivity of lymphadenectomy with consideration of the number of patients depending on number of removed lymph nodes was visualized according to Abdollah et al. [13] (“Abdollah et al.”), the development cohort by Kluth et al. [14] (“Kluth et al. (Dev.)”) and the validation cohort by Kluth et al. [14] (“Kluth et al. (Val.)”) and Rieken et al. [15] (“Rieken et al.”))

Arithmetic mean of sensitivity of correctly identified positive nodes by patient number was estimated according to three datasets, Fig. 1.

Abdollah et al. [13]:

$$\mathrm{f}\left(\mathrm{nLN}\right)=\text{sensitivity}\left(\mathrm{nLN}\right)$$

is dependent on the number of removed lymph nodes (nLN) and Kluth et al. [14] (development and validation cohort) and Rieken et al. [15]:

$$1-\textit{sensitivity}=1-f(nLN)$$

**Fig. 2:**
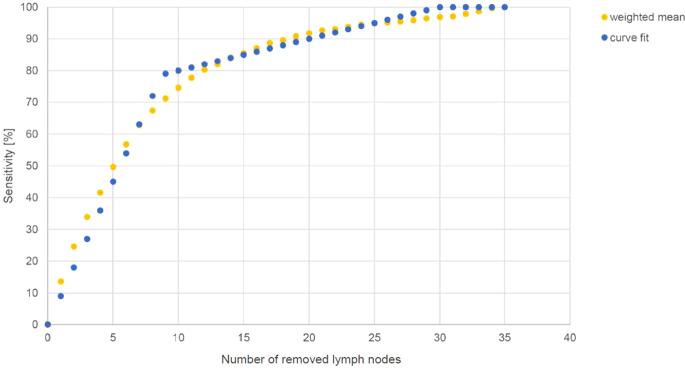
Curve fit of weighted mean sensitivity (fit to the weighted mean sensitivity [“weighted mean”] by Abdollah et al. [13], Kluth et al. [14] and Rieken et al. [15]: f(nLN) = 9 × nLN / 100 for 0 ≤ nLN ≤ 8 and f(nLN) = (nLN + 70) / 100 for 9 ≤ nLN ≤ 29 and f(nLN) = 1 for nLN ≥ 30)

The best fit was derived for the sensitivity f(nLN), Fig. 2. Three formulations were developed depending on the number of removed nodes:

**(II): Sensitivity levels **for the procedure to detect correctly node-positive disease

$$f(nLN)=9 \times nLN/100$$

for 0 ≤ nLN ≤ 8,

$$f(nLN)=(nLN+70)/100$$

for 9 ≤ nLN ≤ 29,

$$f(nLN)=1$$

for nLN ≥ 30.

A commercial optimization approach of sensitivity for linear approximation with two domains of nLK (minimal least squares, solver function, Excel) reveals too complicated parameters for a rule of thumb (nLK 0 − 7.20 : 8.27 × nLK + 5.90; nLK 7.21 − 29.98 : 1.09 × nLK + 67.41; nLK > 29.98 : 1) and our formulation has less least square value than a rounding of the solver’s solution.

### Application of the model

Due to the inclusion of the preoperative estimations of the prevalence of lymph node metastasis, the formulation (I) integrates the usual risk factors: cT stage or GS or preoperative PSA or number of positive cores, dependent on the applied estimation.

For example, the

$$npv=1-\text{prevalence}$$

for f(nLN) = 0 and

$$npv=1$$

for f(nLN) = 1 are resulting in plausible boundary conditions.

**Fig. 3:**
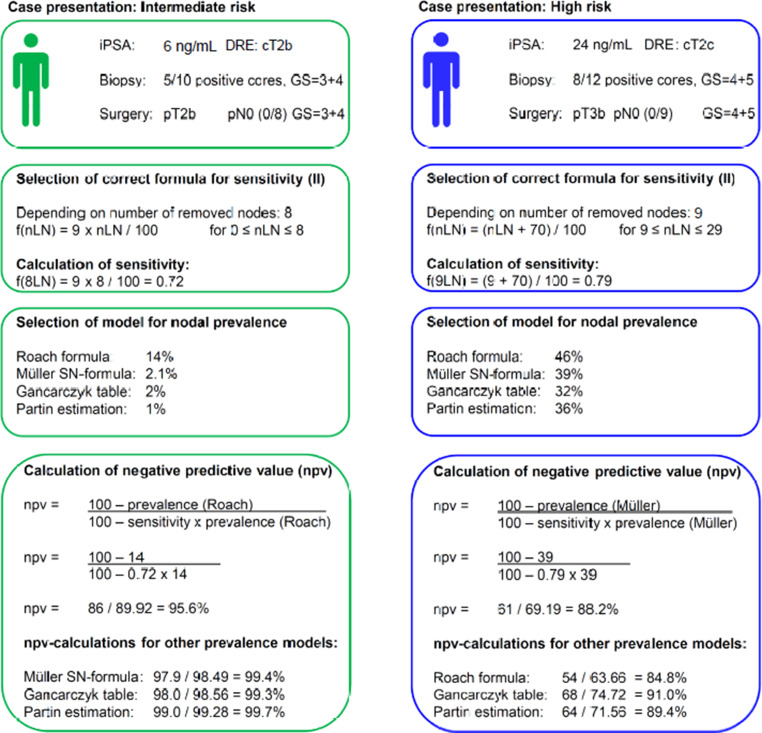
Example calculation (an intermediate and a high-risk example were given to demonstrate similarities and differences between prevalence models and the formula and to show its application. In the first step the correct sensitivity formula (II) is chosen depending on the number of removed nodes. In a second step, the prevalence of positive nodes is calculated based on the preferred model and available parameters. Third, the npv (I) is calculated to assess the need for adjuvant treatment)

**Fig. 4:**
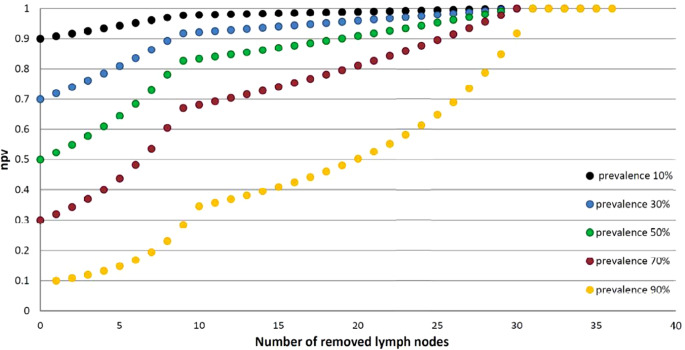
Prevalence-dependent npv (npv was demonstrated as function from prevalence (10–90%) and number of removed lymph nodes according to the developed assessment tool and sensitivity levels)

For easier comprehension, the use of both formulations (I and II) with given prevalence models is demonstrated in Fig. 3. At first, the sensitivity formula (II) is chosen depending on the number of removed nodes. In a second step, the prevalence of positive nodes is calculated based on the preferred model. Third, the npv (I) is calculated to assess the need for adjuvant treatment. Fig. 4 demonstrates the npv dependent on the number of removed lymph nodes for given prevalence levels of lymph nodes metastases.

### Comparison with other models

A comparison of the formula with available models was performed. Detailed exemplary calculations of npv in typical clinical constellations are shown using the Roach (Table 2), Gancarczyk (Table 3), or Partin estimations (Table 4) of N1 prevalence [9–11].

**Table 2:** With the initial formulation (I) of the negative predictive value and, e.g., use of the Roach calculation for estimation of the prevalence of lymph node metastasis, the following values are calculated [11]

PSA	GS	Prevalence according to Roach et al. in %	nLN	(II) sensitivity	(I) npv
5	6	3.3	5	0.45	0.981
5	6	3.3	15	0.85	0.995
> 10^a^	6	7.3	15	0.85	0.988
> 10^a^	9	37.3	5	0.45	0.753
5	8	23.3	5	0.45	0.857
> 10^a^	8	27.3	10	0.8	0.930
> 20^b^	8	33.3	10	0.8	0.907
> 20^b^	8	33.3	15	0.85	0.928
> 20^b^	8	33.3	20	0.9	0.951
25	10	56.7	5	0.45	0.582

*GS* Gleason score, *PSA* prostate-specific antigen in ng/ml, *nLN* number of removed lymph nodes, *npv* negative predictive value

^a^> 10: 11

^b^> 20: 21

**Table 3:** With the initial formulation (I) of the negative predictive value and, e.g., use of the Gancarczyk calculation for estimation of the prevalence of lymph node metastasis, the following values are calculated [10]

PSA	GS	Positive cores (%)	Prevalence according to Gancarczyk et al. in %	nLN	(II) sensitivity	(I) npv
5	6	45	2	5	0.45	0.989
5	6	45	2	15	0.85	0.997
> 10^a^	6	25	4	15	0.85	0.994
> 10^a^	9	25	8	5	0.45	0.954
5	8	65	9	5	0.45	0.948
> 10^a^	8	65	14	10	0.8	0.961
> 20^b^	8	65	32	10	0.8	0.914
> 20^b^	8	65	32	15	0.85	0.934
> 20^b^	8	65	32	20	0.9	0.955
25	10	65	32	5	0.45	0.794

*GS* Gleason score, *PSA* prostate-specific antigen in ng/ml, *nLN* number of removed lymph nodes, *npv* negative predictive value

^a^> 10: 11

^b^> 20: 21

**Table 4:** With the initial formulation (I) of the negative predictive value and, e.g., use of the Partin calculation for estimation of the prevalence of lymph node metastasis the following values are calculated [9]

PSA	GS	T stage	Prevalence according to Partin/Eifler et al. in %	nLN	(II) sensitivity	(I) npv
5	6	2b	1	5	0.45	0.994
5	6	2b	1	15	0.85	0.998
> 10^a^	6	2b	2	15	0.85	0.997
> 10^a^	9	2b	36	5	0.45	0.764
5	8	2b	11	5	0.45	0.936
> 10^a^	8	1c	8	10	0.8	0.983
> 20^b^	8	1c	8	10	0.8	0.983
> 20^b^	8	1c	8	15	0.85	0.987
> 20^b^	8	1c	8	20	0.9	0.991
25	10	2a	22	5	0.45	0.866

*GS* Gleason score, *PSA* prostate-specific antigen in ng/ml, *nLN* number of removed lymph nodes, *npv* negative predictive value

^a^> 10: 11

^b^> 20: 21

Kluth et al. [14] developed a so-called pathologic (postoperative) nodal staging score (pNSS) which represents the probability of correctly being staged as node negative, dependent on the number of removed nodes and different patient characteristics. The pT stage or GS or PSA were applied for an estimation of the pathological nodal staging score. The calculated npv is compared with our formulation in Table 5. Our formulation gives higher estimations of the npv, especially in high-risk situations.

**Table 5:** Comparison of npv from Kluth et al. [14] with pathologically derived parameters with our formulation with different estimated prevalences

T stage	GS	PSA	nLN	Kluth npv (%)	(I) (npv Roach) (%)	(I) (npv Briganti) (%)	(I) (npv Partin) (%)	(I) (npv mean) (%)	(I) npv mean/Kluth npv
2a	6	8	5	96.7 (T, GS, PSA)	97	98.3	100	98.4	1.018
2c	7a	10	5	99 (T)	90.1	97.8	97.8	95.2	0.961
2c	7a	10	12	99.6 (T)	96.5	99.3	99.3	98.3	0.987
3a	7a	10	5	88.6 (T)	90.1	91.2	–*	90.6	1.023
3a	7a	10	12	94.5 (T)	96.5	96.9	–*	96.7	1.023
2a	9	10	5	61.3 (GS)	75.8	91.2	94.2	87.1	1.421
2a	9	10	12	71.7 (GS)	90.6	96.9	98.0	95.2	1.328
2a	7a	5	5	94.6 (PSA)	92.2	98.3	98.9	96.5	1.020
2a	7a	5	12	97.4 (PSA)	97.3	99.4	99.6	98.8	1.015
2a	7a	15	5	83.7 (PSA)	87.9	97.8	97.8	94.5	1.129
2a	7a	15	12	92.3 (PSA)	95.7	99.3	99.3	98.1	1.063

*no T3a stage available in the Partin tables

*nLN* number of removed lymph nodes, *GS* Gleason score, pT stage (cT stage in our formulation) [14], *PSA* prostate-specific antigen in ng/ml, PSA = 10 ng/ml and GS = 7 and T = 2b and number of positive cores = 0.33, if not otherwise specified. *Kluth* mean of development and validation cohort [14], Roach [11], Briganti [8], Partin [9], *nLN* number of removed lymph nodes, *npv* negative predictive value

## Discussion

In order to develop a decision-support model for an assessment of the individual benefit of pelvic lymph node irradiation in the postoperative setting of prostate cancer, we constructed in a theoretical mathematical derivation based on published clinical data a simple *formula to estimate true node negativity* depending on the number of removed nodes and the preoperative prevalence of lymph node metastasis for histopathologically pN0 prostate cancer patients. The formulation integrates risk factors for estimation of the prevalence of lymph node metastasis. The prevalence model can be selected depending on clinical indication/practice and patient selection. The formula is helpful to assess, in an experienced clinical context, a patient’s risk after a negative lymphadenectomy (pN0) to guide adjuvant treatment. Therefore, Fig. 4 can be easily used as a clinical decision tool, needing only the number of removed nodes and the calculated preoperative prevalence of node positivity to determine the remaining risk for nodal involvement. Aiming to find a smart assessment tool, we preferred a linear relationship between the number of removed lymph nodes and sensitivity with only two domains of nLN. To test the formula applicable as a rule of thumb, we estimated the difference between the mathematically calculated approach and the mean of published sensitivity curves. The deviation of the rule of thumb is in the worst case in the range of not more than 8% (maximal 7.8% for the lowest removed node number, i.e., nLN = 9).

Several reports deal with *missed lymph nodes* in the treatment of prostate cancer. False-negative surgical results or geographical miss in radiotherapy are described [17]. In 34 patients, 13% of 91 pathologic lymph node metastases were found outside a standard lymphadenectomy field [18] and in 65.6% of 61 patients, 30.2% sentinel lymph node localizations were outside a standard pelvic irradiation volume [19]. ELND leads to higher rates of pN+ stages (10–24.1%) compared to standard dissection (0–5.2%) [20]. Müller et al. described a 94% nodal clearance rate in a high-risk cohort with combined pelvic irradiation and ADT when potential regions of target miss, i.e., individual sentinel nodes, were included in radiation treatment volumes [21]. No advantage for T1 and T2 tumors, but a 50% reduction of lymphatic recurrences in T3 tumors was reported after adequately dosed pelvic irradiation [22]. Pan et al. found a statistically significant benefit in disease-specific survival after pelvic irradiation in their retrospective series in comparison to radiotherapy to the prostate/seminal vesicles alone [23]. PET/CT might be able to visualize nodal lesions. In a series of 39 patients with rising PSA after radical prostatectomy (pT2–3a pN0), lymph node metastases were found in 13 patients [24]. However, the detection rate (focal uptake interpreted as tumor) of the best currently available PET tracer (PSMA) is limited at low PSA levels, indicating the relevance of the derived assessment tool for adjuvant needs (i.e., PSA level below 0.2 ng/mL: detection rate in meta-analysis 0.4 [25]).

In an analysis of the National Cancer Data Base in 7225 patients with pN1-prostate cancer, adjuvant radiotherapy combined with hormonal therapy significantly decreased the risk of death (5-year overall survival 88.8%) compared to no adjuvant treatment (85.2%), whereas hormonal (82.9%) or radiotherapy (88.3%) alone did not [26]. However, *prospective randomized trials failed* to show a benefit for whole-pelvis irradiation, potentially due to patient selection [1]. However, lymphadenectomy or pelvic radiotherapy in intermediate- or high-risk prostate cancer should encompass more than the standard volumes. Holl et al. described 6% false-negative findings in 2020 patients receiving a sentinel node-based lymphadenectomy [27]. Our formulation reveals a wider range, especially at lower values.

In special situations, postoperative elective irradiation of the pelvic lymph nodes might be indicated, e.g., for postoperative elevation of prostate-specific antigen (PSA) with faster PSA velocity. Therefore, an estimation of the probability of lymph node metastasis might be useful. With the above-derived formula, the urologist and the radiation oncologist can easily rate this risk. Clinical experience and consideration of usual criteria such as PSA velocity still have to be considered and the decision to treat the pelvic lymph nodes must not be based only on such a mathematical model, but has to be supported by clinical experience.

The derived formulations are based on *three main assumptions*. Firstly, the probability of false positivity of histopathological examination is negligible. In common cases, prostate cancer is easily identified in histological analysis by hematoxylin and eosin staining. In more difficult and questionable cases, immunohistochemical stains are available to confirm the prostatic origin [28]. Therefore, in every entity (not only in prostate cancer) with a negligible false-positive rate, the derived formulation (I) of a negative predictive value npv = p(N0IpN0) can be used with the assumptions of prevalence and sensitivity dependent on a clinical marker like the number of removed lymph nodes f(nLN) and can also be used for other malignancies. Regarding prostate cancer, more complex immunohistochemical or molecular examination is able to improve the detection of micrometastases in pN0 stages [29, 30]. This might further enhance the incidence of pN+ stages compared to the used pathological strategies. Therefore, the derived formula is created for usual histological approaches.

Secondly, the underlying figures of the publications by Abdollah et al. and Kluth et al. might not show true sensitivity due to their analytical approaches [13, 14]. However, data by Kluth et al. were externally validated [15]. These three datasets describe dependencies between the sensitivity of lymph node metastasis in pN0 stage and the number of removed lymph nodes and were used for further analysis. The amount of the mean value between the curves is driven by the large number of patients in the validation by Rieken et al. and, to a lesser extent, in data of Abdollah et al., causing the resulting mean of the curve f(nLN). There might be some overlap between patients in the publications. However, the large number of patients and similar derived curves (Fig. 1) support the usefulness of our developed formulation of the negative predictive value dependent on the number of removed nodes. As seen in Fig. 2, the approximation ((II): f(nLN)) leads to slight compensation of the values found by Abdollah et al. [13].

Thirdly, false-negative histological examinations might be related to limited lymphadenectomy not encompassing positive nodes. This risk decreases with increased number of removed nodes [13, 14, 31, 32]. In addition, significantly higher rates of biochemical relapse correlated with molecular N1 stage in histopathologic pN0-staged samples and were reclassified as molecular N1 in 29% of pN0 patients [33]. In line with these findings, Pagliarulo et al. described immunohistochemical detection of 13.3% occult lymph node metastases in 180 pT3 pN0-staged prostate cancers, indicating that standard H&E workup results in a false-negative rate due to sampling error and/or lower sensitivity of conventional light microscopy [3]. These occultly involved nodes led to a similar prognosis compared to standard histological pN1 stage. Immunohistochemistry might also have increased the amount of pN1 in the publications used. The described higher rate of relapse and the rate of 13–29% immunohistochemically found N+-stages might therefore lead to an underestimation of the derived npv estimations.

Comparable results of our formula were observed to the clinical nodal staging score by Kluth et al. [32] applying preoperative parameters. The pathological score by Kluth et al. [14] differed most in high-risk constellations, potentially due to the use of a single parameter or the use of postoperative findings [14].

In the examples, the dependence of the negative predictive value from prevalence and number of removed lymph nodes is obvious: the higher the prevalence or the lower the number of removed nodes, the lower the npv. The achieved rates of npv are high, except in cases of very high prevalence and low nLN. This is plausible and in line with other reports [14]. In cases with a high forecasted prevalence, a pN0 stage is less probable. This was shown with regard to the required number of examined lymph nodes for adequate nodal staging, which depended on pT stage, GS, resection margins, and preoperative PSA [15].

Most published prevalence values were dependent on features such as PSA level, GS, number of positive cores, or T‑stage [8–12]. It is essential to estimate the prevalence dependent on the used parameters. For example, preoperative PSA and GS were used by the Roach estimation, while the percentage of positive cores and T stage were applied by the Müller formula [11, 12].

Limitations of the developed formula comprise retrospective data of large patient cohorts and the absence of advanced pathological (immunohistochemistry) or imaging modalities (preoperative MRI to avoid geographical miss during lymphadenectomy). The use of published datasets instead of own patient cohorts might be a remarkable limitation for the accuracy of the calculated individual lymphatic risk after pN0 diagnosis. A clinical validation of the derived formula in a large cohort of patients seems to be difficult: for a validation, all patients in a cohort being pN0 after surgery would have to be followed without initiation of antihormonal treatment. In case of stable PSA, a true N0 situation is possible after a defined timepoint after surgery. In case of rising PSA, an involvement of lymph nodes has to be proven, maybe by surgical removement, PET (e.g., PSMA), MRI, or CT. If imaging doesn’t find a positive lymph node metastasis, maybe it was too early for the lymph node examination. PSMA-positive lymph nodes might be very small or may be otherwise false positive. Therefore, because of this number of uncertainties in every individual patient in this imaginary cohort, we have chosen a way of using published sensitivities and a theoretical derivation for the rule of thumb, considering the context of clinical experience for every patient. However, current changes of guidelines led to the recommendation to perform PSMA-PET/CT in cases of PSA elevation after surgery. A comparison of such a PSMA-PET/CT-cohort with regard to development of detected small PSMA-PET-positive lymph nodes might serve as a clinical test of the formulation in the future. The calculated magnitudes of npv and the error estimation should allow application in an experienced setting for the individual patient.

## Conclusion

A formulation was defined to give the clinician—as a smart assessment tool—a rule of thumb as to whether adjuvant treatment for the risk of undetected pelvic lymph nodes in surgically staged prostate cancer patients is needed. Clinical circumstances have to be respected for the final decision in the individual patient.

## References

[1] Dirix P, Joniau S, Van den Bergh L, Isebaert S, Oyen R, Deroose CM, Lerut E, Haustermans K (2014). The role of elective pelvic radiotherapy in clinically node-negative prostate cancer: a systematic review. Radiother Oncol.

[2] Abdollah F, Gandaglia G, Suardi N, Capitanio U, Salonia A, Nini A, Moschini M, Sun M, Karakiewicz PI, Shariat SF, Montorsi F, Briganti A (2015). More extensive pelvic lymph node dissection improves survival in patients with node-positive prostate cancer. Eur Urol.

[3] Pagliarulo V, Hawes D, Brands FH, Groshen S, Cai J, Stein JP, Lieskovsky G, Skinner DG, Cote RJ (2006). Detection of occult lymph node metastases in locally advanced node-negative prostate cancer. J Clin Oncol.

[4] Briganti A, Karnes RJ, Da Pozzo LF, Cozzarini C, Capitanio U, Gallina A, Suardi N, Bianchi M, Tutolo M, Salonia A, Di Muzio N, Rigatti P, Montorsi F, Blute M (2011). Combination of adjuvant hormonal and radiation therapy significantly prolongs survival of patients with pT2–4 pN+ prostate cancer: results of a matched analysis. Eur Urol.

[5] Carrie C, Hasbini A, de Laroche G, Richaud P, Guerif S, Latorzeff I, Supiot S, Bosset M, Lagrange JL, Beckendorf V, Lesaunier F, Dubray B, Wagner JP, N’Guyen TD, Suchaud JP, Crehange G, Barbier N, Habibian M, Ferlay C, Fourneret P, Ruffion A, Dussart S (2016). Salvage radiotherapy with or without short-term hormone therapy for rising prostate-specific antigen concentration after radical prostatectomy (GETUG-AFU 16): a randomised, multicentre, open-label phase 3 trial. Lancet Oncol.

[6] Ghadjar P, Hayoz S, Bernhard J, Zwahlen DR, Holscher T, Gut P, Guckenberger M, Hildebrandt G, Müller AC, Plasswilm L, Papachristofilou A, Stalder L, Biaggi-Rudolf C, Sumila M, Kranzbuhler H, Najafi Y, Ost P, Azinwi NC, Reuter C, Bodis S, Kaouthar K, Wust P, Thalmann GN, Aebersold DM (2015). Acute toxicity and quality of life after dose-intensified salvage radiation therapy for biochemically recurrent prostate cancer after prostatectomy: first results of the randomized trial SAKK 09/10. J Clin Oncol.

[7] Ghadjar P, Hayoz S, Bernhard J, Zwahlen DR, Stein J, Holscher T, Gut P, Polat B, Hildebrandt G, Müller AC, Putora PM, Papachristofilou A, Schär C, Dal Pra A, Biaggi RC, Wust P, Aebersold DM, Thalmann GN (2018). Impact of dose intensified salvage radiation therapy on urinary continence recovery after radical prostatectomy: results of the randomized trial SAKK 09/10. Radiother Oncol.

[8] Briganti A, Larcher A, Abdollah F, Capitanio U, Gallina A, Suardi N, Bianchi M, Sun M, Freschi M, Salonia A, Karakiewicz PI, Rigatti P, Montorsi F (2012). Updated nomogram predicting lymph node invasion in patients with prostate cancer undergoing extended pelvic lymph node dissection: the essential importance of percentage of positive cores. Eur Urol.

[9] Eifler JB, Feng Z, Lin BM, Partin MT, Humphreys EB, Han M, Epstein JI, Walsh PC, Trock BJ, Partin AW (2013). An updated prostate cancer staging nomogram (Partin tables) based on cases from 2006 to 2011. BJU Int.

[10] Gancarczyk KJ, Wu H, McLeod DG, Kane C, Kusuda L, Lance R, Herring J, Foley J, Baldwin D, Bishoff JT, Soderdahl D, Moul JW (2003). Using the percentage of biopsy cores positive for cancer, pretreatment PSA, and highest biopsy Gleason sum to predict pathologic stage after radical prostatectomy: the Center for Prostate Disease Research nomograms. Urology.

[11] Roach M, Marquez C, Yuo HS, Narayan P, Coleman L, Nseyo UO, Navvab Z, Carroll PR (1994). Predicting the risk of lymph node involvement using the pre-treatment prostate specific antigen and Gleason score in men with clinically localized prostate cancer. Int J Radiat Oncol Biol Phys.

[12] Müller AC, Zips D, Ernst A, Bares R, Martus P, Weckermann D, Schilling D, Bedke J, Stenzl A (2017). OC-0127: Individualized prediction of nodal involvement based on sentinel-node dissection of prostate cancer. Radiother Oncol.

[13] Abdollah F, Sun M, Thuret R, Jeldres C, Tian Z, Briganti A, Shariat SF, Perrotte P, Montorsi F, Karakiewicz PI (2012). Lymph node count threshold for optimal pelvic lymph node staging in prostate cancer. Int J Urol.

[14] Kluth LA, Abdollah F, Xylinas E, Rieken M, Fajkovic H, Sun M, Karakiewicz PI, Seitz C, Schramek P, Herman MP, Becker A, Loidl W, Pummer K, Nonis A, Lee RK, Lotan Y, Scherr DS, Seiler D, Chun FK, Graefen M, Tewari A, Gonen M, Montorsi F, Shariat SF, Briganti A (2014). Pathologic nodal staging scores in patients treated with radical prostatectomy: a postoperative decision tool. Eur Urol.

[15] Rieken M, Kluth LA, Seitz C, Abufaraj M, Foerster B, Mathieu R, Karakiewicz PI, Bachmann A, Briganti A, Roupre M, Gonen M, Shariat SF, Seebacher V (2017). External validation of the pathologic nodal staging score for prostate cancer: a population-based study. Clin Genitourin Cancer.

[16] Altman DG (1995). Practical statistics for medical research.

[17] Schwenck J, Olthof SC, Pfannenberg C, Reischl G, Wegener D, Marzec J, Bedke J, Stenzl A, Nikolaou K, la Fougère C, Zips D, Müller AC (2019). Intention to treat analysis of (68)Ga-PSMA and (11)C-choline PET/CT versus CT for prostate cancer recurrences after surgery. J Nucl Med.

[18] Joniau S, Van den Bergh L, Lerut E, Deroose CM, Haustermans K, Oyen R, Budiharto T, Ameye F, Bogaerts K, Van Poppel H (2013). Mapping of pelvic lymph node metastases in prostate cancer. Eur Urol.

[19] Ganswindt U, Schilling D, Müller AC, Bares R, Bartenstein P, Belka C (2011). Distribution of prostate sentinel nodes: a SPECT-derived anatomic atlas. Int J Radiat Oncol Biol Phys.

[20] Li R, Petros FG, Kukreja JB, Williams SB, Davis JW (2016). Current technique and results for extended pelvic lymph node dissection during robot-assisted radical prostatectomy. Investig Clin Urol.

[21] Müller AC, Eckert F, Paulsen F, Zips D, Stenzl A, Schilling D, Alber M, Bares R, Martus P, Weckermann D, Belka C, Ganswindt U (2016). Nodal clearance rate and long-term efficacy of individualized sentinel node-based pelvic intensity modulated radiation therapy for high-risk prostate cancer. Int J Radiat Oncol Biol Phys.

[22] Perez CA, Michalski J, Brown KC, Lockett MA (1996). Nonrandomized evaluation of pelvic lymph node irradiation in localized carcinoma of the prostate. Int J Radiat Oncol Biol Phys.

[23] Pan CC, Kim KY, Taylor JM, McLaughlin PW, Sandler HM (2002). Influence of 3D-CRT pelvic irradiation on outcome in prostate cancer treated with external beam radiotherapy. Int J Radiat Oncol Biol Phys.

[24] Henkenberens C, Derlin T, Bengel FM, Ross TL, Wester HJ, Hueper K, Kuczyk MA, Christiansen H, von Klot CA (2018). Patterns of relapse as determined by (68)Ga-PSMA ligand PET/CT after radical prostatectomy: importance for tailoring and individualizing treatment. Strahlenther Onkol.

[25] Hope TA, Goodman JZ, Allen IE, Calais J, Fendler WP, Carroll PR (2019). Metaanalysis of (68)Ga-PSMA-11 PET accuracy for the detection of prostate cancer validated by histopathology. J Nucl Med.

[26] Wong AT, Schwartz D, Osborn V, Safdieh J, Weiner J, Schreiber D (2016). Adjuvant radiation with hormonal therapy is associated with improved survival in men with pathologically involved lymph nodes after radical surgery for prostate cancer. Urol Oncol.

[27] Holl G, Dorn R, Wengenmair H, Weckermann D, Sciuk J (2009). Validation of sentinel lymph node dissection in prostate cancer: experience in more than 2,000 patients. Eur J Nucl Med Mol Imaging.

[28] Epstein JI, Egevad L, Humphrey PA, Montironi R (2014). Best practices recommendations in the application of immunohistochemistry in the prostate: report from the International Society of Urologic Pathology consensus conference. Am J Surg Pathol.

[29] Lunger L, Retz M, Bandur M, Souchay M, Vitzthum E, Jäger M, Weirich G, Schuster T, Autenrieth M, Kübler H, Maurer T, Thalgott M, Herkommer K, Koll F, Gschwend JE, Nawroth R, Heck MM (2020). KLK3 and TMPRSS2 for molecular lymph-node staging in prostate cancer patients undergoing radical prostatectomy. Prostate Cancer Prostatic Dis.

[30] Maxeiner A, Grevendieck A, Pross T, Rudl M, Arnold A, Stephan C, Jung K, Miller K, Kilic E, Busch J (2019). Lymphatic micrometastases predict biochemical recurrence in patients undergoing radical prostatectomy and pelvic lymph node dissection for prostate cancer. Aktuelle Urol.

[31] Briganti A, Chun FK, Salonia A, Gallina A, Zanni G, Scattoni V, Valiquette L, Rigatti P, Montorsi F, Karakiewicz PI (2007). Critical assessment of ideal nodal yield at pelvic lymphadenectomy to accurately diagnose prostate cancer nodal metastasis in patients undergoing radical retropubic prostatectomy. Urology.

[32] Kluth LA, Abdollah F, Xylinas E, Rieken M, Fajkovic H, Seitz C, Sun M, Karakiewicz PI, Schramek P, Herman MP, Becker A, Hansen J, Ehdaie B, Loidl W, Pummer K, Lee RK, Lotan Y, Scherr DS, Seiler D, Ahyai SA, Chun FK, Graefen M, Tewari A, Nonis A, Bachmann A, Montorsi F, Gonen M, Briganti A, Shariat SF (2014). Clinical nodal staging scores for prostate cancer: a proposal for preoperative risk assessment. Br J Cancer.

[33] Heck MM, Retz M, Bandur M, Souchay M, Vitzthum E, Weirich G, Schuster T, Autenrieth M, Kubler H, Maurer T, Thalgott M, Herkommer K, Gschwend JE, Nawroth R (2018). Molecular lymph node status for prognostic stratification of prostate cancer patients undergoing radical prostatectomy with extended pelvic lymph node dissection. Clin Cancer Res.

